# Statin shapes inflamed tumor microenvironment and enhances immune checkpoint blockade in non–small cell lung cancer

**DOI:** 10.1172/jci.insight.161940

**Published:** 2022-09-22

**Authors:** Wenjun Mao, Yun Cai, Danrong Chen, Guanyu Jiang, Yongrui Xu, Ruo Chen, Fengxu Wang, Xuehai Wang, Mingfeng Zheng, Xinyuan Zhao, Jie Mei

**Affiliations:** 1 Department of Cardiothoracic Surgery, The Affiliated Wuxi People’s Hospital of Nanjing Medical University, Wuxi, China.; 2 Wuxi College of Clinical Medicine,; 3 State Key Laboratory of Reproductive Medicine, Center for Global Health, School of Public Health, and; 4 Key Laboratory of Modern Toxicology of Ministry of Education, School of Public Health, Nanjing Medical University, Nanjing, China.; 5 Department of Occupational Medicine and Environmental Toxicology, Nantong Key Laboratory of Environmental Toxicology, School of Public Health, Nantong University, Nantong, China.; 6 Department of Oncology, The Affiliated Wuxi People’s Hospital of Nanjing Medical University, Wuxi, China.

**Keywords:** Metabolism, Oncology, Bioinformatics, Cancer immunotherapy

## Abstract

Immune checkpoint blockade (ICB) therapy has achieved breakthroughs in the treatment of advanced non–small cell lung cancer (NSCLC). Nevertheless, the low response due to immuno-cold (i.e., tumors with limited tumor-infiltrating lymphocytes) tumor microenvironment (TME) largely limits the application of ICB therapy. Based on the glycolytic/cholesterol synthesis axis, a stratification framework for EGFR-WT NSCLC was developed to summarize the metabolic features of immuno-cold and immuno-hot tumors. The cholesterol subgroup displays the worst prognosis in immuno-cold NSCLC, with significant enrichment of the cholesterol gene signature, indicating that targeting cholesterol synthesis is essential for the therapy for immuno-cold NSCLC. Statin, the inhibitor for cholesterol synthesis, can suppress the aggressiveness of NSCLC in vitro and in vivo and can also drastically reverse the phenotype of immuno-cold to an inflamed phenotype in vivo. This change led to a higher response to ICB therapy. Moreover, both our in-house data and meta-analysis further support that statin can significantly enhance ICB efficacy. In terms of preliminary mechanisms, statin could transcriptionally inhibit PD-L1 expression and induce ferroptosis in NSCLC cells. Overall, we reveal the significance of cholesterol synthesis in NSCLC and demonstrate the improved therapeutic efficacy of ICB in combination with statin. These findings could provide a clinical insight to treat NSCLC patients with immuno-cold tumors.

## Graphical abstract

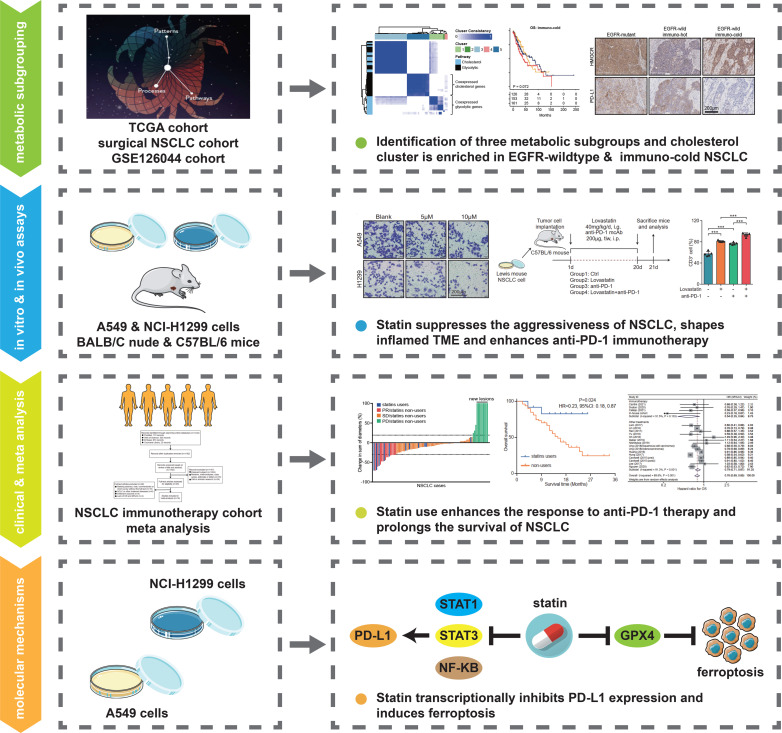

## Introduction

Lung cancer is one of the most widespread cancers worldwide, causing the majority of tumor-induced deaths (1). Non–small cell lung cancer (NSCLC), the most proportional subtype of lung cancer, accounts for more than 85% of total diagnoses (2). Under the widespread application of low-dose computed tomography, a large proportion of NSCLC cases are being diagnosed at earlier stages, greatly improving long-term prognoses (3). Despite this, many patients who progress to advanced stages of NSCLC still face a lack of effective treatment options and a correspondingly negative prognosis. Recent advancements in molecular profiling of NSCLC have contributed to the control of advanced diseases and provide powerful guidance for potentially new therapeutic strategies, such as EGFR tyrosine kinase inhibitors (TKIs) (4). EGFR-TKIs reversibly bind the kinase domain of EGFR, inhibiting activation of downstream signaling pathways (5). Driven by these developments, patients with EGFR-mutated NSCLC have achieved better progression-free survival (PFS) and overall survival (OS). However, a greater proportion of patients does not exhibit targetable mutations in oncogenic driver genes.

In the last decade, immune checkpoint inhibitors (ICIs), particularly for the PD-1/PD-L1 inhibitors, have changed the therapeutic landscape of NSCLC (6). For patients harboring WT EGFR and overexpressed PD-L1, immune checkpoint blockade (ICB) is the preferred therapeutic strategy due to encouraging clinical outcomes (7). ICB and/or ICB plus chemotherapy have been used as alternative selections for first- or second-line treatment of patients with advanced NSCLC (8). Tumor-infiltrating lymphocytes (TILs) and PD-L1 expression are regarded as effective biomarkers for immunotherapy (9, 10). According to the abundance and spatial distribution of TILs, tumors could be discriminated into inflamed, excluded, and deserted subtypes (11). Inflamed tumors are considered to be immuno-hot, whereas both excluded and deserted subtypes are considered to be immuno-cold (12). The former typically responds well to immunotherapy treatment using checkpoint inhibitors (13).

Metabolic reprogramming is recognized as one of the hallmarks of cancer (14). Alterations of metabolic characteristics in the tumor microenvironment (TME) are recognized as important tools for cancer cells that help them interact with the infiltrating immune cells within the TME (15). Tumor cells featured with the Warburg effect can shift glucose metabolism toward aerobic glycolysis, providing cancer cells with energy to promote tumor growth, invasion, and metastasis (16). Recent studies reveal that the inhibition of glucose metabolism can enhance ICB efficacy (17, 18). In addition, cholesterol is the major sterol in mammalian cell membranes, maintaining cell integrity and fluidity and forming intracellular homeostasis; furthermore, enhanced cholesterol biosynthesis promotes cancer cell growth and immune evasion (19). Overall, increasing evidence suggests that metabolic reprogramming can contribute to tumor progression by modulating the TME.

Although the application of ICB has greatly improved the prognosis of those with immuno-hot tumors, a large proportion of NSCLC patients with EGFR WT and immuno-cold tumors still lack effective treatment strategies. Herein, we developed a stratification framework for EGFR-WT NSCLC to summarize the metabolic features of immuno-cold and immuno-hot tumors based on the glycolytic/cholesterol synthesis axis, and we demonstrate that immuno-cold NSCLC exhibited significant enrichment of the cholesterol gene signature. Correspondingly, statin significantly suppressed NSCLC progression and mediated a conversion from an immuno-cold tumor to an inflamed phenotype. Both our in-house data and meta-analysis implicate that statin could significantly enhance the response to immunotherapy. Overall, these results may provide a novel clinical insight for the control of NSCLC using a combination therapy of statin and ICB.

## Results

### Identification of 3 metabolic subgroups based on glycolytic/cholesterol cluster in EGFR-WT NSCLC.

**Figure 1:**
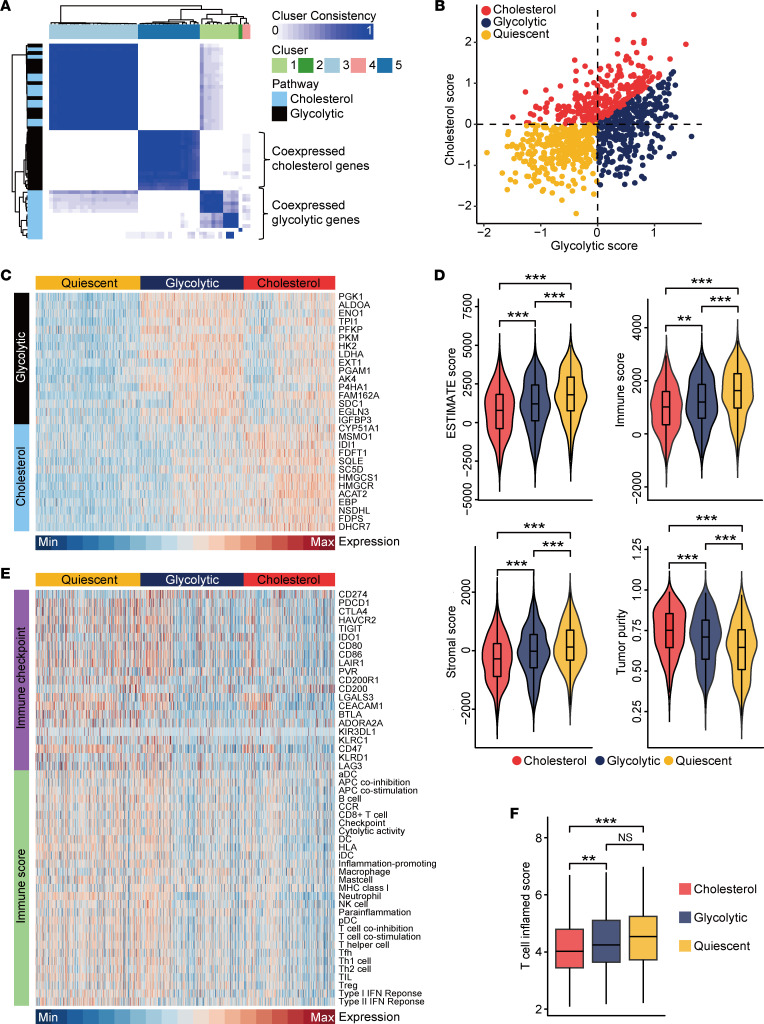
Stratification of EGFR-WT NSCLC samples according to the expression of glycolytic and cholesterol genes. (**A**) Heatmap exhibiting consensus clustering solution for glycolytic and cholesterol genes in EGFR-WT NSCLC samples. (**B**) Scatter plot exhibiting median expression levels of coexpressed glycolytic (*x* axis) and cholesterol (*y* axis) genes in each sample. Metabolic subgroups were appointed according to the relative expression levels of glycolytic and cholesterol genes. (**C**) Heatmap showing expression levels of coexpressed glycolytic and cholesterol genes in 3 metabolic subgroups. (**D**) Violin plot showing 4 TME scores estimated using the ESTIMATE algorithm in quiescent (*n* = 308), glycolytic (*n* = 304), and cholesterol (*n* = 269) subgroups. Data are presented as mean ± SD. Significance was calculated with 1-way ANOVA with Tukey’s multiple-comparison test. **P < 0.01, ***P < 0.001. (**E**) Heatmap showing immune checkpoints expressions and levels of immune cells and immune-related response in 3 subgroups. (**F**) Box plot showing T cell inflamed score in quiescent (*n* = 308), glycolytic (*n* = 304), and cholesterol (*n* = 269) subgroups. Data are presented as mean ± SD. Significance was calculated with 1-way ANOVA with Tukey’s multiple-comparison test. **P < 0.01, ***P < 0.001.

To stratify EGFR-WT NSCLC tumors based on the expression levels of glycolytic and cholesterol genes, we utilized RNA-Seq data from The Cancer Genome Atlas (TCGA) data set (https://xenabrowser.net/datapages/). To select genes coregulated within each pathway, we used consensus clustering to identify 2 groups of coexpressed glycolytic (*n* = 16) and cholesterol (*n* = 13) genes, which were utilized for metabolic subtyping (Figure 1A). Median expression levels of coexpressed glycolytic and cholesterol genes were calculated for each sample and utilized in assigning 1 of 3 main metabolic profiles specifically relevant to these 2 pathways: glycolytic (highly expressed glycolytic genes, low expression of cholesterol genes), cholesterol (low expression of glycolytic genes, highly expressed cholesterol genes), and quiescent (low expression of both glycolytic and cholesterol genes) (Figure 1B). Expression levels of glycolytic and cholesterol genes across the metabolic subgroups were visualized as Figure 1C.

Previous research has revealed that metabolic grouping is associated with clinical and mutational profiles in multiple cancer types (20, 21). We therefore sought to characterize the clinical relevance of the previously described metabolic subtypes in EGFR-WT NSCLC. The result shows that the metabolic subtypes were significantly related to T stage, N stage, and TNM stage (Supplemental Figure 2A; supplemental material available online with this article; https://doi.org/10.1172/jci.insight.161940DS1), in addition to a statistical association between metabolic subtypes and prognoses (Supplemental Figure 2B). Furthermore, we observed that metabolic subtypes were significantly correlated with the frequency of copy number variation (CNVs) (Supplemental Figure 3, A–F) and gene mutations (Supplemental Figure 4, A–F) of several critical functional genes in NSCLC, including TP53, TTN, ZFHX4, CSMD3, SYNE1, and ROS1. In short, tumors with glycolytic and cholesterol phenotypes exhibited more advanced stages, poorer prognoses, and more frequent genetic alterations compared with those of the quiescent phenotype.

Next, we investigated the difference in immunofactors across metabolic subtypes. Tumors with the cholesterol phenotype showed the lowest ESTIMATE score (22), immune score, and stromal score, while exhibiting the highest tumor purity (Figure 1D). Moreover, immune checkpoint expressions, most immune cell populations, and the activities of multiple immune-related pathways were low in tumors with cholesterol phenotypes (Figure 1E). Furthermore, evidence revealed that the lowest T cell inflamed score was observed in the tumors with cholesterol phenotype (Figure 1F). Overall, our findings indicate that cholesterol subtypes coincide significantly with immuno-cold phenotypes.

### Cholesterol cluster predicts poor prognosis and is enriched in EGFR-WT & immuno-cold NSCLC.

**Figure 2:**
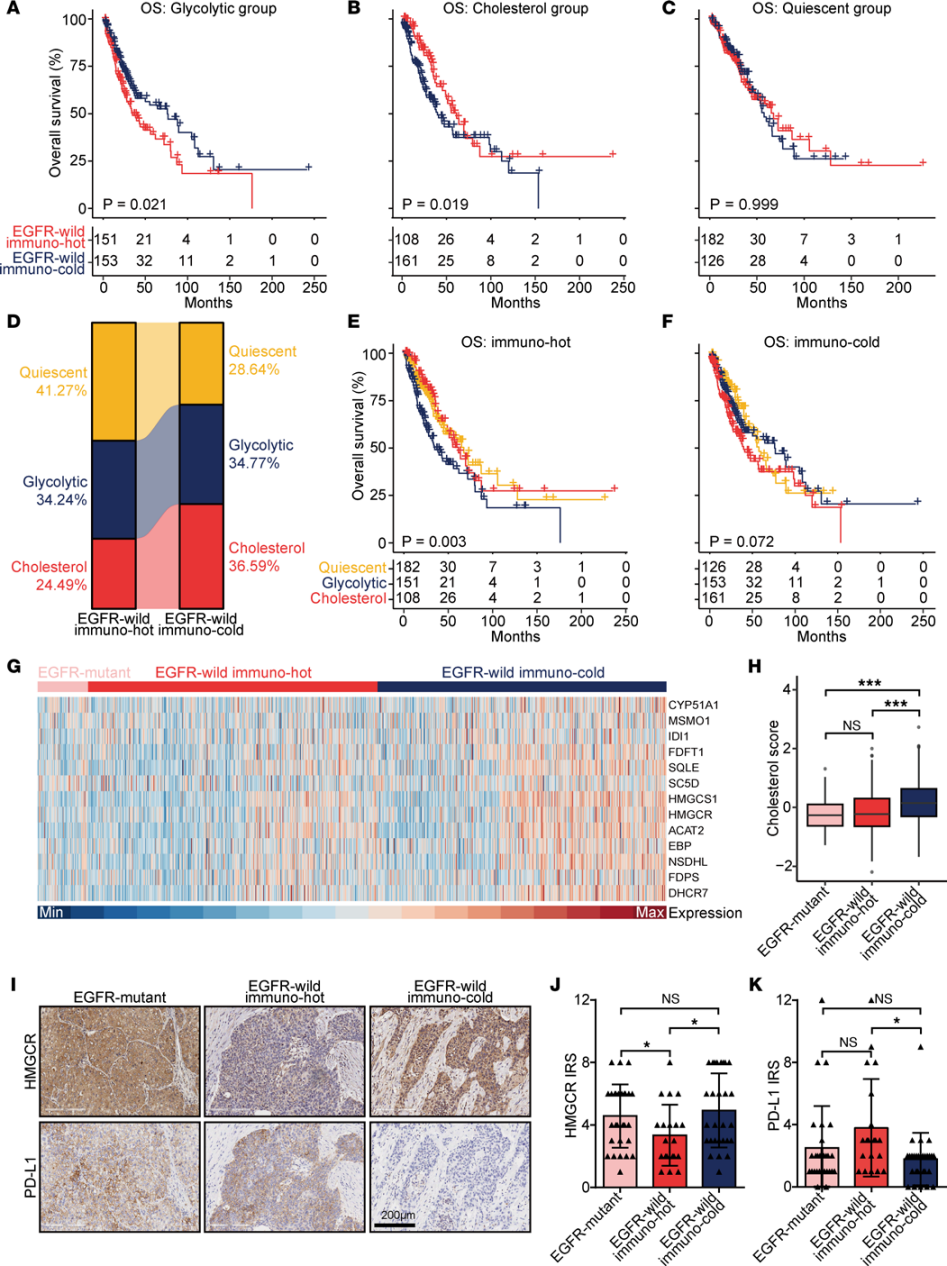
Clinical impacts of immunophenotypes in EGFR-WT NSCLC samples with various metabolic subtypes. (**A**–**C**) Kaplan-Meier survival analysis of EGFR-WT NSCLC patients with log-rank test in various metabolic subtypes stratified by T cell inflamed score. Immuno-hot subtype was defined with high T cell inflamed score, and the immuno-cold subtype was defined with low T cell inflamed score. (**D**) The proportion of various metabolic subtypes in immuno-hot and immuno-cold subtypes. (**E** and **F**) Kaplan-Meier survival analysis of EGFR-WT NSCLC patients with log-rank test in immuno-hot and immuno-cold subtypes stratified by metabolic subtypes. (**G**) Heatmap showing expression levels of coexpressed cholesterol genes in EGFR-mutant, EGFR-WT & immuno-hot, EGFR-WT & immuno-cold NSCLC samples. (**H**) Box plot showing cholesterol score in EGFR-mutant (*n* = 77), EGFR-WT & immuno-hot (*n* = 441), and EGFR-WT & immuno-cold NSCLC (*n* = 440) groups. Data are presented as mean ± SD. Significance was calculated with 1-way ANOVA with Tukey’s multiple-comparison test. ***P < 0.001. (**I**–**K**) Representative images showing HMGCR and PD-L1 expression in EGFR-mutant (*n* = 28), EGFR-WT & immuno-hot (*n* = 20), and EGFR-WT & immuno-cold NSCLC (*n* = 28) groups, along with semi-quantitative analysis. Total original magnification, 200×. Data are presented as mean ± SD. Significance was calculated with 1-way ANOVA with Tukey’s multiple-comparison test for **J**, and Kruskal-Wallis with Dunn’s multiple-comparison test for **K**. *P < 0.05.

Given the discrepancy in the antitumor immune status of the 3 metabolic subtypes, we divided EGFR-WT NSCLC samples into immune-hot and immuno-cold subtypes according to the median level of T cell inflamed score. Next, we investigated the impact of immune phenotype across all 3 metabolic subtypes. Survival analyses of clinical patients show that an immune-hot phenotype was an indicator for poor prognosis in the glycolytic subtype (Figure 2A), while predicting a favorable prognosis in the cholesterol subtype (Figure 2B). Notably, the immune phenotypes had no impact on prognosis in the quiescent subtype (Figure 2C). We next compared the distribution of 3 metabolic subtypes in immuno-hot and immuno-cold tumors. The result showed that the cholesterol subtype accounted for a higher percentage of immuno-cold tumors (Figure 2D). Survival analysis showed that high expression levels of glycolytic genes were associated with an unfavorable prognosis for samples with the immuno-hot phenotype, while high expression levels of cholesterol synthesis genes were associated with an unfavorable prognosis for samples with the immuno-cold phenotype (Figure 2, E and F).

We subsequently investigated the expression of cholesterol genes in NSCLC samples with EGFR mutation, EGFR-WT & immuno-hot, and EGFR-WT & immuno-cold. The results show that EGFR-WT & immuno-cold tumors exhibited the highest levels of cholesterol gene expression (Figure 2, G and H). This result was corroborated by our in-house cohort (Figure 2I). HMGCR, the rate-limiting enzyme in cholesterol synthesis, was highly expressed in immuno-cold tumors compared with immuno-hot tumors, and no difference was observed between EGFR-mutant and immuno-cold tumors (Figure 2J). In addition, PD-L1 expression showed the opposite difference (Figure 2K). Moreover, tumors with poor response to anti–PD-1 immunotherapy exhibited significantly higher cholesterol gene expression and cholesterol scores in the GSE126044 data set (Supplemental Figure 5, A and B). Altogether, these results uncover that the cholesterol cluster was highly expressed in immuno-cold tumors and predicts poor prognosis, indicating the interference of cholesterol synthesis could be a promising therapy in EGFR-WT & immuno-cold NSCLC.

### Statin suppresses the aggressiveness of NSCLC in vitro and in vivo.

**Figure 3:**
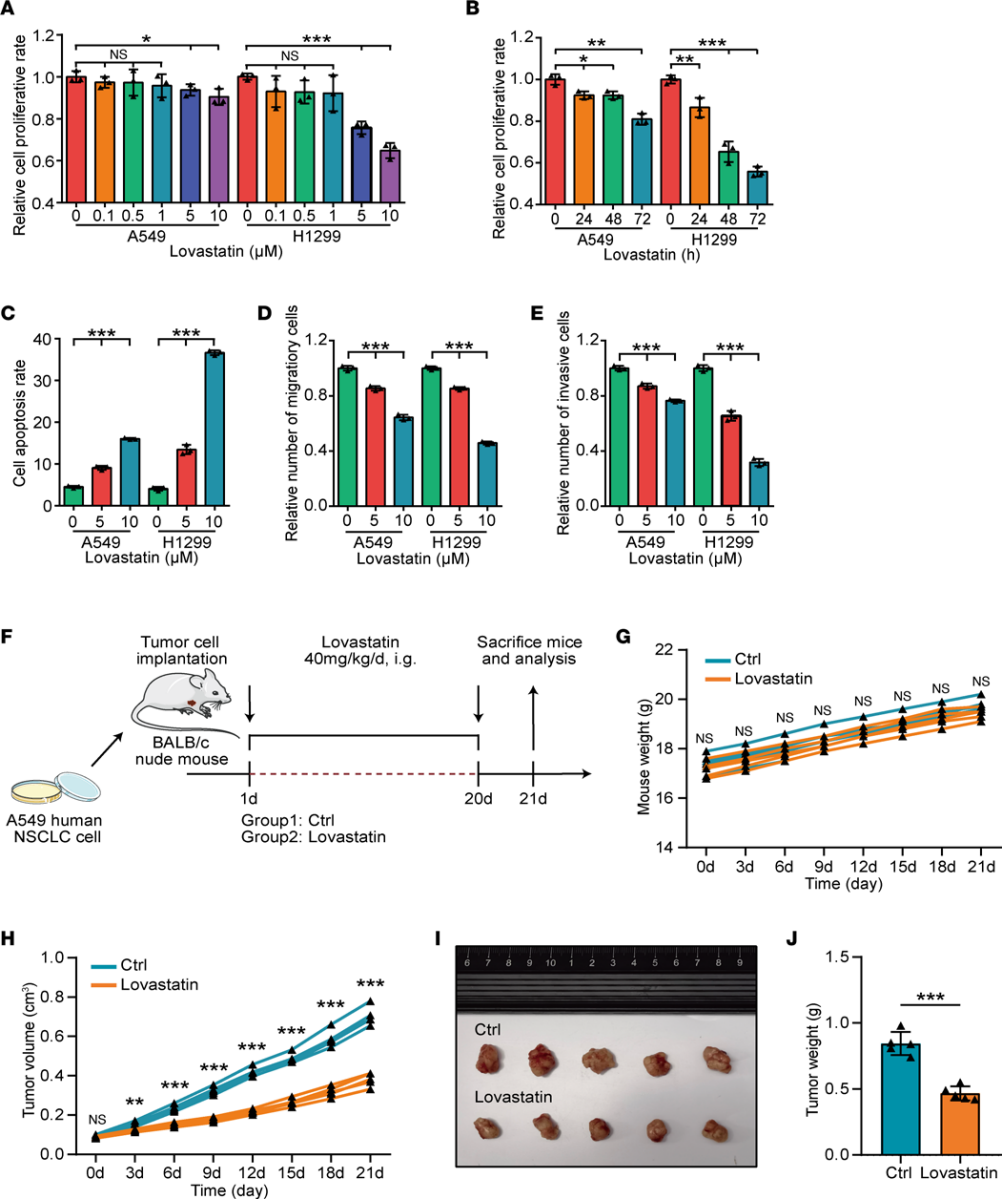
Statin inhibits NSCLC progression in vitro and in nude mouse model. (**A** and **B**) The proliferative capacity of control and statin-treated NSCLC cells was investigated by CCK-8 assay. Dose-dependent antitumor efficacy of statin (10 μM) (**A**); time-dependent antitumor efficacy of statin (24 hours) (**B**). The experiment was performed 3 times. Data are presented as mean ± SD. Significance was calculated with 1-way ANOVA with Tukey’s multiple-comparison test. *P <0.05, **P < 0.01, ***P < 0.001. (**C**) The apoptotic levels of control and statin-treated (5/10 μM, 72 hours) NSCLC cells was investigated by flow cytometry assay. The experiment was performed 3 times. Data are presented as mean ± SD. Significance was calculated with 1-way ANOVA with Tukey’s multiple-comparison test. ***P < 0.001. (**D** and **E**) The migratory and invasive capacities of control and statin-treated (5/10 μM, 72 hours) NSCLC cells were investigated by Boyden chamber assay. The experiment was performed 3 times. Data are presented as mean ± SD. Significance was calculated with 1-way ANOVA with Tukey’s multiple-comparison test. ***P < 0.001. (**F**) Schematic protocol of the antitumor therapy using lovastatin in immunodeficient BALB/c nude mice. (**G**) Weight change curve of mice treated with PBS (*n* = 5) or lovastatin (*n* = 5). Significance was calculated with Student’s *t* test. (**H**) Tumor growth curve of mice treated with PBS (*n* = 5) or lovastatin (*n* = 5). Significance was calculated with Student’s *t* test. **P < 0.01, ***P < 0.001. (**I**) Representative images showing the tumors harvested from mice bearing A549 cells treated with PBS or lovastatin. (**J**) Weight of the harvested tumors (*n* = 5). Data presented as mean ± SD. Significance was calculated with Student’s *t* test. ***P < 0.001.

Statin is a specific inhibitor of the mevalonate (MVA) pathway, which is used to block cholesterol synthesis. In addition, statin acts as tumor-suppressive roles in multiple cancers (22, 24). We selected A549 and NCI-H1299 NSCLC cells to validate the functional roles of statin in EGFR-WT NSCLC. First, a CCK-8 assay uncovered that the antiproliferative effect of statin in the 2 cell lines was both time dependent and dose dependent (Figure 3, A and B). Thus, a dose of 5 or 10 μM and a 72-hour action time were used for subsequent assays. Compared with control cells, statin-treated A549 and NCI-H1299 cells exhibited enhanced apoptotic levels (Figure 3C and Supplemental Figure 6A). In addition, treatment with statin significantly suppressed the migratory and invasive capacities of NSCLC cells (Figure 3, D and E, and Supplemental Figure 6, B and C). Further in vivo assays were also conducted using nude mice bearing A549 cells and lovastatin (Figure 3F). The injection of lovastatin did not affect the weight of the mice (Figure 3G), indicating good tolerance. Predictably, the injection of lovastatin significantly decreased both tumor volumes (Figure 3H) and tumor weight (Figure 3, I and J). Collectively, statin remarkably impedes the progression of NSCLC, which could be a therapeutic drug.

### Statin shapes inflamed TME and enhances anti–PD-1 immunotherapy.

**Figure 4:**
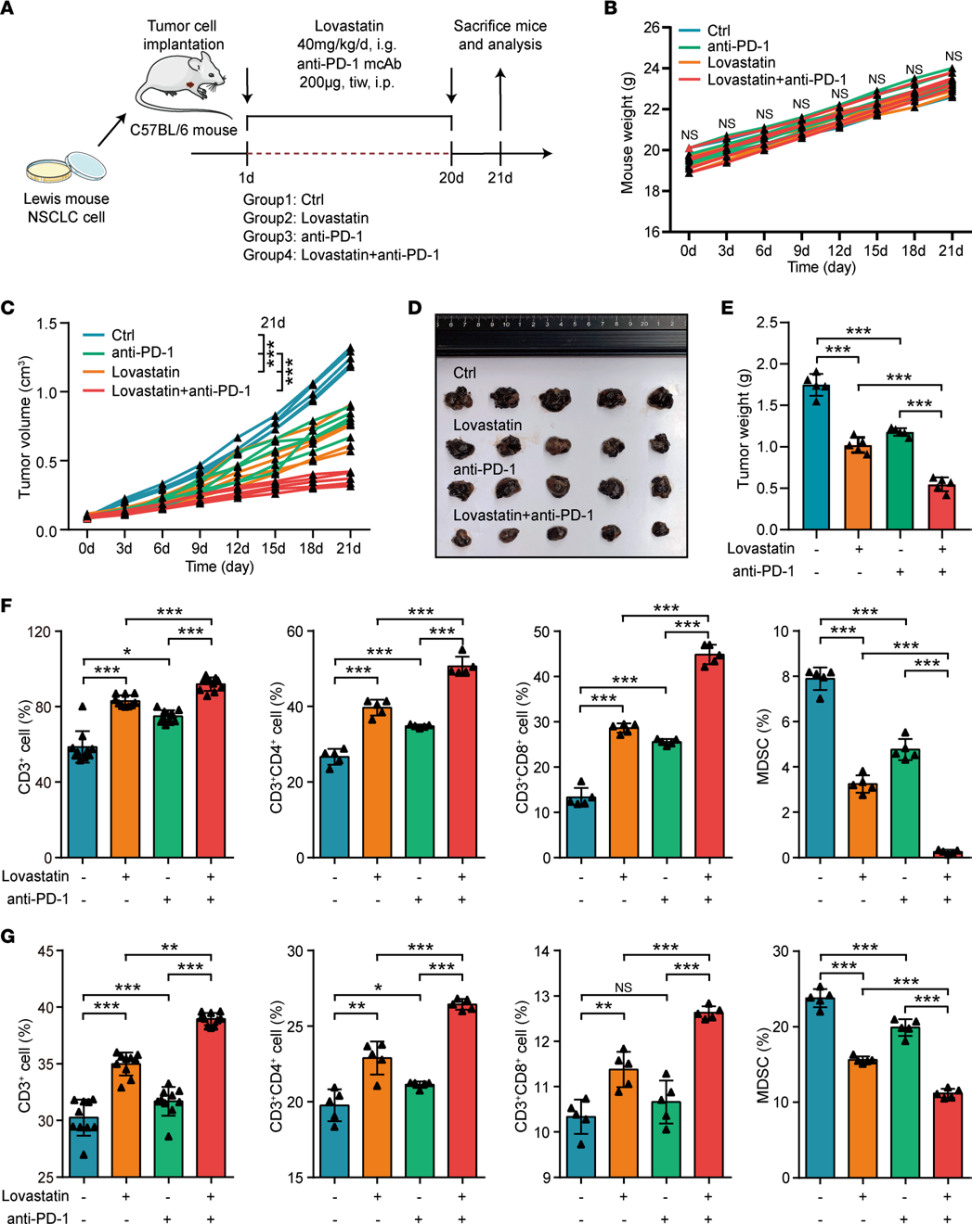
Statin shapes the inflamed TME and enhances immunotherapy. (**A**) Schematic protocol of the combination of anti–PD-1 antibody and lovastatin therapy in immunocompetent C57BL/6 mice. (**B**) Weight change curve of mice treated with PBS (*n* = 5), lovastatin (*n* = 5), anti–PD-1 antibody (*n* = 5), and the combination (*n* = 5). Significance was calculated with 1-way ANOVA with Tukey’s multiple-comparison test. (**C**) Tumor growth curve of mice treated with PBS (*n* = 5), lovastatin (*n* = 5), anti–PD-1 antibody (*n* = 5), and the combination (*n* = 5). Significance was calculated with 1-way ANOVA with Tukey’s multiple-comparison test. ***P < 0.001. (**D**) Representative images showing the tumors harvested from mice bearing Lewis cells treated with PBS, lovastatin, anti–PD-1 antibody, and the combination. (**E**) Weight of the harvested tumors (*n* = 5). Data are presented as mean ± SD. Significance was calculated with 1-way ANOVA with Tukey’s multiple-comparison test. *P < 0.05, ***P < 0.001. (**F**) Quantification of the percent of CD3^+^ total T cells (*n* = 10, since CD3/CD4 and CD3/CD8 were independently labeled), CD3^+^CD8^+^ T cells (*n* = 5), CD3^+^CD4^+^ T cells (*n* = 5), and CD11b^+^Gr-1^+^ MDSC (*n* = 5) in the tumors. Data are presented as mean ± SD. Significance was calculated with 1-way ANOVA with Tukey’s multiple-comparison test. *P < 0.05, ***P < 0.001. (**G**) Quantification of the percent of CD3^+^ total T cells (*n* = 10, since CD3/CD4 and CD3/CD8 were independently labeled), CD3^+^CD8^+^ T cells (*n* = 5), CD3^+^CD4^+^ T cells (*n* = 5), and CD11b^+^Gr-1^+^ MDSC (*n* = 5) in the spleens. Data are presented as mean ± SD. Significance was calculated with 1-way ANOVA with Tukey’s multiple-comparison test. *P < 0.05, **P < 0.01, ***P < 0.001.

Given that immuno-cold tumors exhibited the higher cholesterol cluster compared with immuno-hot tumors, we hypothesized that statin use may increase the therapeutic efficacy of anti–PD-1 immunotherapy. We administrated lovastatin and/or anti–PD-1 antibody to C57BL/6 mice harboring Lewis cells (Figure 4A). Monotherapy or combination therapy did not affect the weight of the mice (Figure 4B), indicating good tolerance. Both tumor volume (Figure 4C) and tumor weight (Figure 4, D and E) were decreased remarkably in mice that received the combined therapy. IHC analysis was conducted to examine the levels of CD45 and CD3. The result showed that lovastatin and combined therapy significantly increased CD45^+^ and CD3^+^ cell infiltration (Supplemental Figure 7, A and B). In addition, flow cytometric analysis uncovered notable elevations in the quantity of total T cells, CD4^+^ T cells, and CD8^+^ T cells, and showed a decrease in the quantity of myeloid-derived suppressor cells (MDSCs) in the tumors and spleens in which both therapies were administered (Figure 4, F and G, and Supplemental Figure 8, A and B). In summary, these findings suggest that statin shapes inflamed TME and raises the efficacy of PD-L1 blockade in vivo.

### Statin use enhances the response to anti–PD-1 therapy and prolongs the survival of NSCLC.

**Figure 5:**
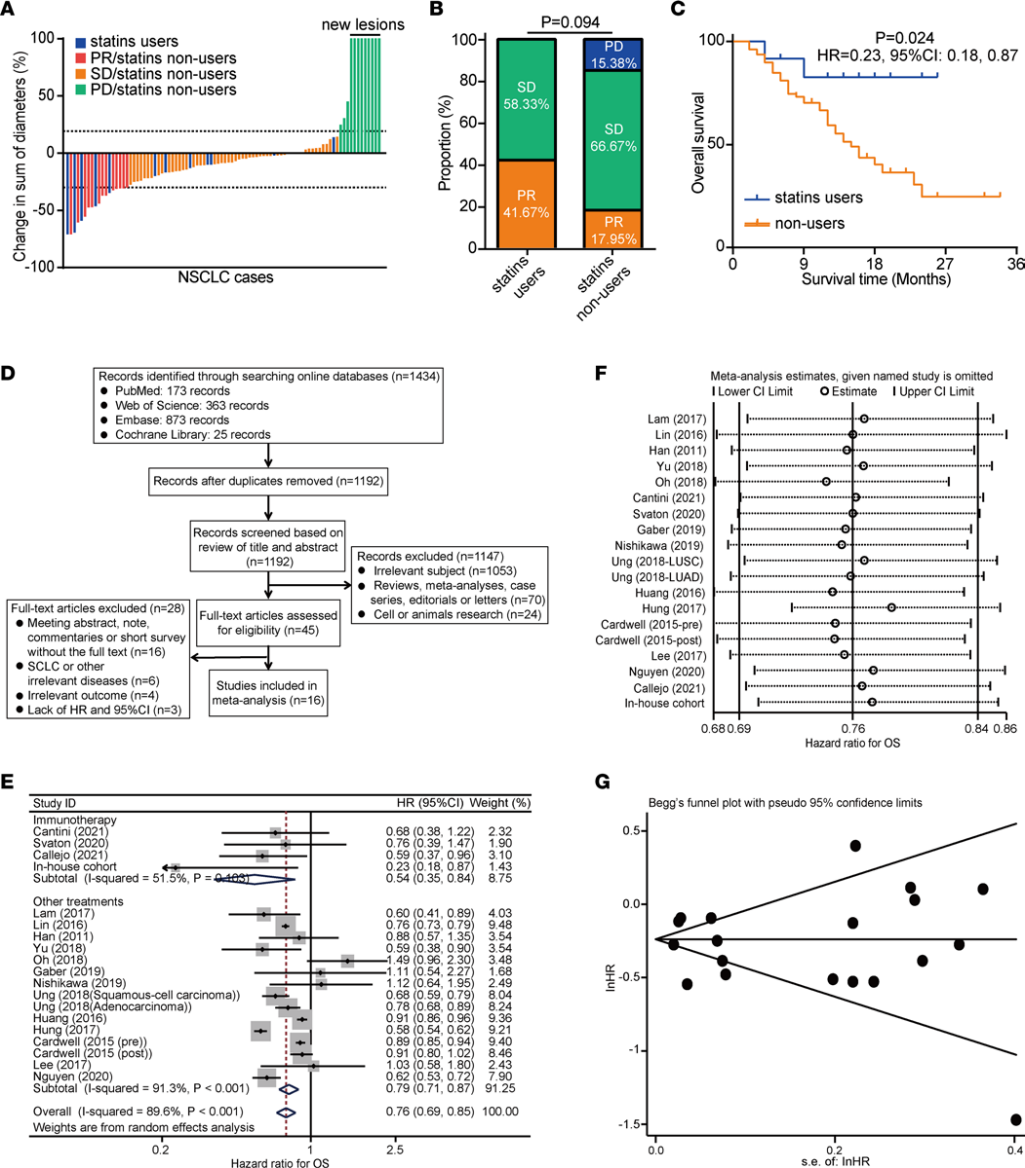
Statin use prolongs the survival of NSCLC. (**A**) Overview of the relative alterations in the sum of diameters in the in-house cohort. PR, partial response; SD, stable disease; PD, progressive disease. (**B**) Comparison of the therapeutic response to immunotherapy in patients with statin use and no statin use in the in-house cohort. Significance was calculated with Pearson’s χ^2^ test. (**C**) Comparison of OS with log-rank test in patients with statin use and those with never statin use in the in-house cohort. (**D**) The flow chart of study selection and inclusion. (**E**) Pooled and subgroup analyses comparing OS in patients with statin use and those with no statin use. (**F**) Sensitivity analysis for the relationship between statin use and OS. (**G**) Funnel graph regarding the relationship between statin use and OS.

Given the strong association between statin and inflamed TME in silico, in vitro, and in vivo, we next examined the effect of statin use on the clinical outcome of NSCLC. We collected an in-house cohort containing 90 NSCLC patients younger than 75 receiving anti–PD-1 immunotherapy, and we evaluated the therapeutic response according to the RECIST 1.1 criteria (Figure 5A). Results indicate that statin use significantly enhanced the therapeutic response to anti–PD-1 immunotherapy and improved OS of NSCLC patients (Figure 5, B and C). Based on the inclusion and exclusion criteria (Figure 5D), 16 studies involving 63,273 participants in total were contained in our meta-analysis, in addition to our in-house cohort (Figure 5D). Overall, statin use was notably related to the improvement of OS, and further analysis indicated a possible association between statin use and improved OS in the subgroup receiving immunotherapy (Figure 5E). Sensitivity analyses were also performed and suggested the reliability of the original studies (Figure 5F). A funnel plot of included studies and Egger’s test revealed no evidence of a publication bias (Figure 5G).

Considering the limited number of NSCLC-focused studies, we performed another meta-analysis on the association between the use of statins in patients receiving ICIs and OS in all cancer types. Six studies involving 2,135 participants were contained, as was our in-house cohort (Supplemental Figure 9A). Statistical analyses suggest that the use of statins in patients with cancer receiving ICIs remarkably improved OS (Supplemental Figure 9B). Sensitivity analyses and publication bias tests were also performed. The results of sensitivity analyses indicate no obvious differences due to the removal of any studies, which suggests the credibility of the included studies (Supplemental Figure 9C), and no publication bias was revealed (Supplemental Figure 9D). Overall, such findings suggest that the use of statins is an auxiliary strategy to raise the response to anti–PD-1 immunotherapy in NSCLC.

### Statin transcriptionally inhibits PD-L1 expression and induces ferroptosis.

Lastly, we investigated the possible mechanisms underlying the statin-induced inflamed phenotype. Overexpression of PD-L1 is a critical mediator of immune escape in many cancer types (25). Thus, we investigated whether statin affected PD-L1 expression. Interestingly, our results indicate that statin significantly downregulated IFN-γ–induced mRNA and protein levels of PD-L1 in vitro (Supplemental Figure 10, A and B) and inhibited PD-L1 in mouse tumor tissues (Supplemental Figure 10C), suggesting that statin inhibited PD-L1 expression at the transcriptional level. We collected 3 classical PD-L1 transcriptional factors, including NF-κB (26), STAT1 (27), and STAT3 (28). Statin did not change IFN-γ–induced total expression but significantly inhibited the phosphorylation of most of these proteins in both A549 and NCI-H1299 cells (Supplemental Figure 10D).

**Figure 6:**
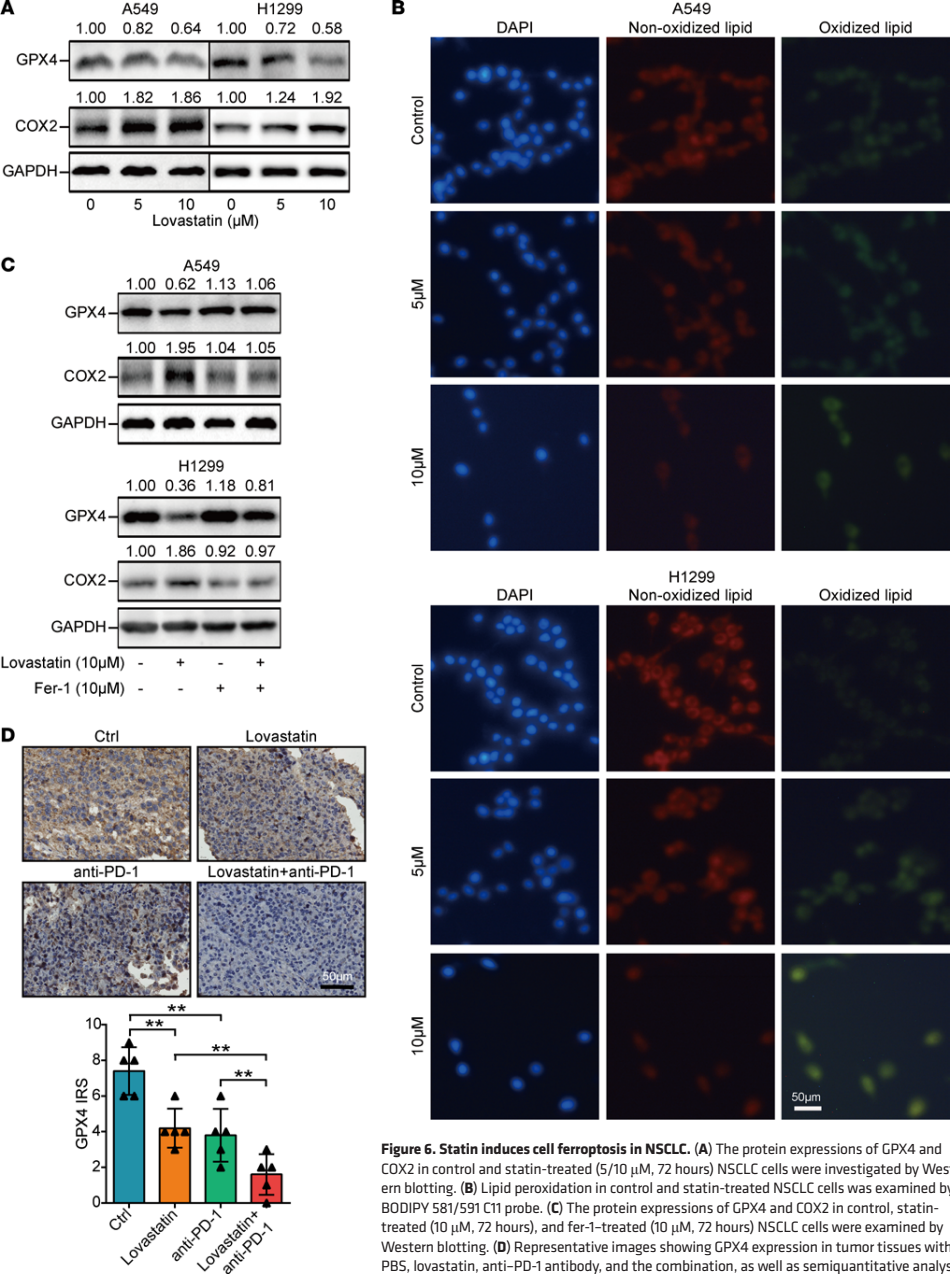
Statin induces cell ferroptosis in NSCLC. (**A**) The protein expressions of GPX4 and COX2 in control and statin-treated (5/10 μM, 72 hours) NSCLC cells were investigated by Western blotting. (**B**) Lipid peroxidation in control and statin-treated NSCLC cells was examined by BODIPY 581/591 C11 probe. (**C**) The protein expressions of GPX4 and COX2 in control, statin-treated (10 μM, 72 hours), and fer-1–treated (10 μM, 72 hours) NSCLC cells were examined by Western blotting. (**D**) Representative images showing GPX4 expression in tumor tissues with PBS, lovastatin, anti–PD-1 antibody, and the combination, as well as semiquantitative analysis (*n* = 5). Total original magnification, 400×. Data are presented as mean ± SD. Significance was calculated with 1-way ANOVA with Tukey’s multiple-comparison test.**P < 0.01.

Wang et al. reported a mechanism by which T cells eliminate tumor cells by inducing tumor cell ferroptosis (29). Thus, tumor cells might inhibit ferroptosis to shape an immuno-cold microenvironment (30). It was uncovered that isopentenyl pyrophosphate regulated the biosynthesis of GPX4 via the MVA pathway and that HMGCR acted as a critical role in the synthesis of isopentenyl pyrophosphate (31). Thus, statin might promote tumor cell ferroptosis. First, we found that ferroptosis suppressors were enriched in several pathways, which included cholesterol homeostasis (Supplemental Figure 11A), suggesting the significant role of cholesterol-related pathways in ferroptosis. In addition, EGFR-WT & immuno-cold tumors exhibited a higher ferroptosis suppressor score but a lower ferroptosis driver score (Supplemental Figure 11, B and C). In NSCLC cells, statin downregulated GPX4 but upregulated COX2 (Figure 6A). In addition, statin increased the oxidized lipid but decreased the nonoxidized lipid (Figure 6B). The inhibitor for ferroptosis, ferrostatin-1 (fer-1), could reverse statin-induced GPX4 downregulation (Figure 6C) and could also reverse statin-induced cell death in NSCLC cells (Supplemental Figure 12, A and B). Furthermore, lovastatin and combined treatment inhibited GPX4 expression in mouse tumor tissues (Figure 6D). Collectively, these data reveal that the downregulation of PD-L1 and the induction of ferroptosis are critical mechanisms underlying statin-mediated inflamed TME.

## Discussion

Increasing evidence has revealed that metabolic reprogramming impacts oncogenesis and the TME (32, 33). Metabolic gene changes and their clinical impacts have been reported in multiple cancer types, indicating that altered metabolism-related genes may serve as promising therapeutic targets in cancer (16, 34). Previous research established a stratification framework for EGFR-WT NSCLC based on the transcriptome integrating PD-L1 expression and metabolic features, and this research tried to investigate the interplay between metabolism and TME (35). However, transcriptional PD-L1 expression could not reflect TME characteristics of EGFR-WT NSCLC. T cell inflamed score based on the expression levels and weighting coefficient of 18 genes could comprehensively describe TME and predict immunotherapeutic efficacy (36). In the current research, we constructed a potentially novel stratification framework for EGFR-WT NSCLC based on the metabolic features and T cell inflamed score.

Glycolytic and cholesterol metabolism are usually altered in cancer cells, which impact tumorigenesis and cancer progression (37, 38). Karasinska et al. constructed a stratification framework for pancreatic cancer based on the glycolytic/cholesterol cluster and found that patients with glycolytic tumors were associated with a worse prognosis in both resectable and metastatic pancreatic cancer (39). The stratification framework was also applied in other cancer types, but different correlations between metabolic subtypes and prognosis were observed in various cancer types (20, 21). These studies reveal that glycolytic/cholesterol metabolic processes play different functional roles in oncogenesis and cancer progression. In our research, the glycolytic/cholesterol cluster was used to stratify EGFR-WT NSCLC, and 3 distinct subgroups were identified. The glycolytic subgroup displayed the worst prognosis in immuno-hot tumors while the cholesterol subgroup showed the worst prognosis in immuno-cold NSCLC. In addition, immuno-cold NSCLC was enriched with upregulated cholesterol genes. As such, these findings revealed that statin may exert a critical pharmacological effect on immuno-cold NSCLC.

Statin is a specific inhibitor of the MVA pathway implicated in cholesterol and has been considered the most effective strategy for the management of high cholesterol (40). In addition to the pharmacodynamic properties regarding hyperlipidemia treatment, the association between statin use and cancer has been widely investigated (24). It has been revealed that dysregulation of the MVA pathway could accelerate tumorigenesis and progression partially mediated by HMGCR as a metabolic oncogene (41, 42). The present research validated the tumor-suppressive roles of statin in NSCLC in vitro and in vivo. Alongside general antitumor effects, statin was reported to modulate the TME (23). Similarly, statin could activate antigen-presenting cells and CD8^+^ T cells in tumor tissues in head and neck carcinoma (43). Moreover, the PD-L1 signaling pathway suppresses the activation of T lymphocytes, enhancing tumorigenesis and immune escape. In this research, we found that statin could suppress the aggressiveness of NSCLC and transcriptionally inhibited PD-L1 expression. In addition, the low level of ferroptosis in tumor cells shapes an immuno-cold microenvironment (30), and the induction of ferroptosis could enhance ICB efficacy (44). Statin could induce ferroptosis in NSCLC via downregulating GPX4. Overall, statin shapes inflamed TME and enhances anti–PD-1 immunotherapy in NSCLC via transcriptionally inhibiting PD-L1 and inducing ferroptosis.

The critical role of statin in regulating the TME indicates that combination therapy with PD-1 inhibitors could be an effective strategy. Based on our in-house cohort, we found that the use of statin was remarkably related to improved clinical outcome in NSCLC patients. Although multiple studies have uncovered the combination of statin with PD-1 inhibitors in various cancers (45–50), these clinical studies had too few patients receiving statin to evaluate potential confounding effects. Therefore, we conducted a meta-analysis to assess the effect of statin use on anti–PD-1 immunotherapy, and we observed a potential indication that the use of statin may improve OS in the NSCLC patients receiving immunotherapy. Given the limited number of studies focusing on NSCLC, we performed another meta-analysis on the association between the use of statin in patients receiving ICIs and OS in all types of cancers. The results suggest that the use of statins in patients with cancer receiving ICIs remarkably improved OS. Thus, these clinical findings provide strong evidence that the use of statins may enhance the response to anti–PD-1 immunotherapy.

As an unavoidable issue, the correlation between the doses of statin used for antitumor therapy and the safe therapeutic doses applied in patients to treat cholesterolemia should not be ignored. The dose of lovastatin used for antitumor study varies from 20 to 100 mg/kg/day (d) in the mouse, which were approximately varies from 1.62 to 8.11 mg/kg/d in the adult according to the dose conversion factor between humans and mice (51–53). In addition, the dose of lovastatin used for antitumor clinical trials varies from 0.33 mg/kg/d to 20 mg/kg/d (adult weight was regarded as 60 kg) (clinicaltrials.org; NCT00853580, NCT04297033, NCT00585052, NCT01478828, NCT00584012). The dose of lovastatin used to reduce blood fat in clinical practice varies from 0.33 to 1.33 mg/kg/d. Overall, we could observe that the dose of lovastatin used to reduce blood fat is lower than the antitumor dose. In this research, we selected 40 mg/kg/d for the in vivo assay (approximately equal to 3.24 mg/kg/d in an adult), which is about twice the maximum lipid-lowering dose but is at the median level of reported antitumor doses. Despite that a high dose of lovastatin was used in the antitumor assay, it did not cause weight loss, indicating the tolerable toxicity. Furthermore, evidence from our in-house cohort and meta-analysis suggest that statins had a sensitizing effect on anti–PD-1 therapy even at clinical lipid-lowering doses. The optimal dose of statins use for antitumor therapy needs to be further explored.

In summary, we first performed systematic bioinformatics using transcriptomic data and ultimately identified cholesterol metabolism as an anticancer target. Based on this, we provided multidimensional evidence to illustrate the critical role of statin in suppressing tumor progression and promoting inflamed TME in NSCLC. Overall, the current study provides a comprehensive rationale for the combination of statins with PD-1 inhibitors in immuno-cold NSCLC.

## Methods

### Acquisition of public data.

The NSCLC standardized RNA-Seq gene expression profiles, clinical annotation, and mutation data of the TCGA data sets were acquired from the UCSC Xena (https://xenabrowser.net/datapages/). Concerning the T cell inflamed score, which was calculated based on the 18 specific genes related to T cell inflammation and their weighting coefficients (36), the top 50% cases were designated as the immuno-hot subgroup, and the rest were assigned to the immuno-cold group. Finally, the EGFR-mutation group included 77 patients, the EGFR-WT & immuno-hot group included 441 patients, and the EGFR-WT & immuno-cold included 440 patients. In addition, the clinical annotation of the TCGA samples were exhibited in Supplemental Table 1. Moreover, the GSE126044 data set, which contained the RNA-Seq data from NSCLC patients before receiving anti–PD-1 immunotherapy, was downloaded from the Gene Expression Omnibus database (https://www.ncbi.nlm.nih.gov/geo/) (54).

### Metabolic subgrouping.

The genes in the gene sets “HALLMARK_CHOLESTEROL_BIOSYNTHESIS” and “HALLMARK_GLYCOLYSIS” in the molecular signatures (MSigDB) database (http://www.gsea-msigdb.org/gsea/msigdb) (55) were used as cholesterol and glycolytic genes, respectively. Consensus clustering was conducted on cholesterol and glycolytic genes utilizing R package ConsensusClusterPlus (paramters: reps = 100, pItem = 0.8, pFeature = 1). Ward.D2 and Spearman distances were utilized as the clustering algorithm and distance metric, respectively, with *k* = 5. Median expression levels of coexpressed glycolytic and cholesterol genes were utilized to discriminate quiescent (glycolytic ≤ 0, cholesterol ≤ 0), glycolytic (glycolytic > 0, glycolytic > cholesterol), and cholesterol (cholesterol > 0, cholesterol > glycolytic) metabolic subgroups to each sample. The quiescent group included 308 patients, the glycolytic group included 304 patients, and the cholesterol included 269 patients. The cholesterol score was calculated by the single-sample gene set enrichment analysis (ssGSEA) utilizing markers of coexpressed cholesterol genes in R package “GSVA” (56).

### Estimation of the immunological features of the TME.

Given that the bulk RNA-Seq data from tumor tissues contain both tumor and normal cells, we assessed the immunological features of the TME of each sample. The ESTIMATE algorithm (22), a method estimating tumor purity and stromal and immune cells from tumor samples based on a bulk transcriptomic profile, was performed to calculate tumor purity, ESTIMATE score, immune score, and stromal score. To further understand the immunological status of each patient, a group of signature genes of 29 immune cells and immune-related pathways (57) was utilized to assess the infiltration levels of various immune cell subsets, and the activities of immune-related pathways and functions of each patient were estimated by utilizing the ssGSEA in R package “GSVA” (56).

### Definition of ferroptosis-associated genes and scores.

The gene lists of ferroptosis drivers and suppressors were acquired from the FerrDb database (http://www.zhounan.org/ferrdb/current/). For GSEA of ferroptosis suppressors, we downloaded the h.all.v7.4.symbols.gmt subclass from the MSigDB (55), and it was used as the background. The enrichment analysis was conducted using the R package “clusterProfiler”. To acquire the results of gene set enrichment, the minimum gene set was set to 5 and the maximum gene was set to 5,000. Terms with *P* ≤ 0.05 were deemed to be statistically significant. In addition, the ferroptosis driver and suppressor scores was of each specimen were calculated by utilizing the ssGSEA in R package “GSVA” (56).

### Reagents and antibodies.

Lovastatin (catalog HY-N0504) was purchased from MedChemExpress. Recombined active IFN-γ protein (catalog KGH2016) was purchased from KeyGEN (Nanjing, China). Mouse spleen lymphocyte separation kit (catalog P8860) and mouse tumor–infiltrating tissue lymphocyte separation kit (catalog P9000) were purchased from Solarbio. Liberase (catalog 5401020001), DNase I (catalog DN25), and type IV Collagenase (catalog C5138) were purchased from MilliporeSigma. The PD-1 in vivo mAb (catalog BE0273) was purchased from BioXCell. The inhibitor for ferroptosis, fer-1 (catalog T6500), was purchased from TargetMol. The BODIPY 581/591 C11 probe (catalog D3861) was purchased from Thermo Fisher Scientific. Antibodies used in the study and their source were exhibited in Supplemental Table 2.

### Clinical samples.

Two independent single-center cohorts were included in the current study. The first clinical cohort included 76 patients with NSCLC from 2016 to 2020 in The Affiliated Wuxi People’s Hospital of Nanjing Medical University. Among these patients, there were 48 patients harboring WT EGFR and 28 patients harboring mutant EGFR. The status of EGFR was detected as previously described (58). The paraffin-embedded tumor samples from these patients were collected for IHC staining. The detailed clinic-pathological characteristics can be found in Supplemental Table 3.

The second clinical cohort included 90 NSCLC patients younger than 75 receiving anti–PD-1 immunotherapy from 2019 to 2021 in our hospital. The therapeutic response was evaluated according to the RECIST 1.1 criteria, which were demarcated into complete response (CR), partial response (PR), stable disease (SD), and progressive disease (PD). The detailed clinic-pathological characteristics and immunotherapy information could be found in Supplemental Table 4.

### IHC staining and semiquantitative assessment.

IHC staining was directly conducted on the above sections following the established procedures. The primary antibodies used in this research were as follows: anti‑HMGCR (1:200 dilution, catalog 13533-1-AP, ProteinTech), anti–PD-L1 (ready-to-use, catalog GT2280, GeneTech), and anti‑CD8 (ready-to-use, catalog GT2112, GeneTech). Antibody staining was visualized with DAB and hematoxylin counterstain, and stained sections were scanned using Aperio Digital Pathology Slide Scanners.

The stained sections were independently assessed by 2 pathologists. Only stained tumor cells were included for semiquantitative assessment of HMGCR and PD-L1 expression according to the criteria of immunoreactivity score (IRS) (59). For semiquantitative assessment of CD8 staining, infiltration level was evaluated by estimating the percentage of cells with membrane staining in the tumor stroma. Tumors were discriminated into 3 phenotypes following the spatial distribution of CD8^+^ T cells, including the inflamed, the excluded, and the deserted subtypes (60). As previously defined, the inflamed subtype is considered to be immuno-hot, and both excluded and deserted subtypes are considered to be immuno-cold (Supplemental Figure 1, A–C).

### Cell lines and cell culture.

A549 (catalog KG007) and NCI-H1299 (catalog KG307) NSCLC cell lines were purchased from KeyGEN. In addition, Lewis (catalog KG070) mouse lung cancer cell line was also purchased from KeyGEN. All these cells were cultured at 37°C with 5% CO_2_. A549 cells were maintained in F12K media supplemented with 10% FBS. NCI-H1299 cells were cultured in RPMI-1640 media supplemented with 10% FBS. Lewis cells were cultured in DMEM supplemented with 10% FBS. Mycoplasma-free cells were used in all experiments. Additionally, human cancer cell lines were recently appraised by short tandem-repeat profiling.

### CCK-8 assay.

A549 and NCI-H1299 cells were digested using 0.25% trypsin for 1 minute and resuspended with media containing 10% FBS. Suspended cells were seeded on a 96-well plate with the cell density adjusted to 3 × 10^4^ cells/mL (100 μL/well) and fostered at 37°C in a constant-temperature incubator with 5% CO_2_. To each well, 10 μL CCK-8 was added, after which the 96-well plate was put in the incubator for 2 hours. The OD value of each well was examined at 450 nm by a microplate reader.

### Cell apoptosis assay.

A549 and NCI-H1299 cells were plated in 60 mm dishes and allowed to attach overnight. Cell apoptosis detection was analyzed using Annexin V-PE/7-AAD kit (catalog KGA1024, KeyGEN). The FACSCalibur flow cytometer (Becton Dickinson) was used to conduct the apoptosis analysis. Data for apoptosis were analyzed using FlowJo7.6 software v7.6.5.

### Boyden chamber assay.

For cell invasion and migration assays, 1 × 10^5^ NSCLC cells in serum-free medium supplemented with 5 mg/mL BSA were inoculated to the upper sides of the modified Boyden chamber (8.0 μm, catalog 3422, Corning). The polycarbonate membranes of Boyden chambers were coated with Matrigel (catalog 356234, BD Biosciences) or not. After 24 hours, the invasive or migratory cells on the lower sides of Boyden chambers were fixed and stained with 0.2% crystal violet. The stained cells were photographed, and 3 microscopic fields per sample were randomly selected for quantification.

### Animal models.

Male BALB/C nude mice (5–6 weeks old) and C57BL/6 mice (5–6 weeks old) were obtained from Shanghai SLAC Laboratory Animal Co. Ltd. The mice were raised in specific pathogen–free grade experimental animal centers and were provided with free access to food and water. The mice were housed at 22°C ± 2°C, with 70% relative humidity under a 12-hour light/dark cycle. To establish the NSCLC model, A549 (1 × 10^7^) and Lewis (1 × 10^7^) cells were s.c. injected into the flanks of BALB/C nude mice and C57BL/6 mice without any medium, respectively. Tumors were monitored and regularly measured with calipers every 2–3 days. Two weeks after seeding A549 cells, and 9 days after seeding Lewis cells, tumors reached about 100 mm^3^ in volume. BALB/C nude mice bearing A549 cells were randomized into 2 various groups (*n* = 5), control and lovastatin groups, and C57BL/6 mice bearing Lewis cells were randomized into 4 various groups (*n* = 5), including control, lovastatin, anti–PD-1 antibody, and lovastatin + anti–PD-1 groups. Lovastatin (catalog HY-N0504) was dissolved in PBS and administered to mice intragastrically at 40 mg/kg daily. The PD-1 in vivo mAb (catalog BE0273, BioXCell) was dissolved in PBS, and 200 μg was administered i.p. 3 times a week. The control group was only administered i.p. with PBS.

Treatment was continued until tumors reached 20 mm along the long axis or until 21 days after treatment initiation. The mice were euthanized with excess 0.5% sodium pentobarbital solution to remove the tumors, which were then photographed and weighted. Removed tumors were submitted for IHC and flow cytometry analysis. Tumors were fixed in 4% paraformaldehyde for 20 minutes and permeabilized with 0.5% Triton X-100 in PBS for 10 minutes. Tumor sections were blocked with BSA 5% for 30 minutes. The sections were incubated with primary antibodies against PD-L1 (1:2000 dilution, catalog 66248-1-Ig, ProteinTech), CD45 (1:1,000 dilution, catalog 20103-1-AP, ProteinTech), CD3 (1:1,000 dilution, catalog 17617-1-AP, ProteinTech), and GPX4 (1:200 dilution, catalog CY6959, Abways) at 4°C overnight and followed with secondary antibodies. Antibody staining was visualized with DAB and hematoxylin counterstain, and stained sections were photographed.

### Flow cytometry analysis.

After the mice were sacrificed, tumors and spleens were removed under sterile conditions for flow cytometry. For tumor tissues pretreatment, after tearing off the membrane, the tissues were mechanically shredded, mixed with PBS and 0.05% type IV Collagenase (catalog C5138, MilliporeSigma), 0.001% Liberase (catalog 5401020001, MilliporeSigma), and DNase I (catalog DN25, MilliporeSigma) until no lumps were visible; the tissues were then filtered. For spleen tissue pretreatment, after tearing off the membrane, the tissues were mechanically shredded and then ground on a 70 μm cell strainer with a 2 mL syringe and washed with PBS to collect cell suspension. Next, the filtered cell suspension was collected, and we added mouse spleen (catalog P8860, Solarbio) or tumor lymphocyte separation solution (catalog P9000, Solarbio). After centrifugation (500*g*, room temperature, 20 minutes), lymphocytes were aspirated and washed. Then, the specimens were stained for surface markers for immune cell subsets. CD3/CD4, CD3/CD8, and GR-1/CD11b were labeled independently. The primary antibodies used were exhibited in Supplemental Table 2. Analysis was performed using the CytoFLEX flow cytometer (Beckman Coulter). Data were analyzed using FlowJo software v7.6.5, and random 1 × 10^4^ cells were exhibited as a dot plot.

### qPCR.

Total RNA of NSCLC cells was extracted using TRIzol reagent (catalog 15596026, Invitrogen). The primers for *PD-L1* and *GAPDH* mRNA reverse transcription were synthesized in KeyGEN. Quantitative PCR (qPCR) was conducted using the One-Step TB Green PrimeScript RT-PCR Kit II (SYBR Green) (catalog RR086B, TaKaRa). Primers used for gene amplification were as follows: PD-L1: (forward) 5′-ACAGCTGAATTGGTCATCCC-3′, (reverse) 5′-TGTCAGTGCTACACCAAGGC-3′; GAPDH: (forward) 5′-AGATCATCAGCAATGCCTCCT-3′, (reverse) 5′-TGAGTCCTTCCACGATACCAA-3′.

### Western blotting analysis.

Preprocessed A549 and NCI-H1299 cells were plated in 35 mm dishes (6 × 10^5^ cells/dish). All cellular proteins were harvested with lysis buffer. SDS-PAGE and Western blotting analysis were performed as standard protocols. The primary antibodies used as follows: GPX4 (1:1,000 dilution, catalog CY6959, Abways), COX2 (1:2,000 dilution, catalog 66351-1-Ig, ProteinTech), PD-L1 (1:1,000 dilution, catalog 66248-1-Ig, ProteinTech), STAT1 (1:1,000 dilution, catalog 14994, CST), p-STAT1 (1:1,000 dilution, catalog 9167, CST), STAT3 (1:1,000 dilution, catalog 9139, CST), p-STAT3 (1:2,000 dilution, catalog 9145, CST), NF-κB (1:1,000 dilution, catalog 8242, CST), p–NF-κB (1:1,000 dilution, catalog 3033, CST), and GAPDH (1:5,000 dilution, catalog 60004-1-Ig, ProteinTech). Protein expression levels were normalized to GAPDH for each sample.

### Lipid peroxides measurement.

To visualize the lipid ROS, NSCLC cells were seeded in 6-well plates and treated with the designated conditions. After treatments, cells were stained with 5 μmol/L BODIPY 581/591 C11 probe in accordance with the manufacturer’s instructions for 20 minutes, followed by staining with DAPI; stained cells were photographed.

### Meta-analysis.

The databases of PubMed (https://pubmed.ncbi.nlm.nih.gov), Embase (https://www.embase.com), Web of Science (https://www.webofscience.com/wos/woscc/basic-search), and Cochrane Library (https://www.cochranelibrary.com/search) were systematically searched up until November 4, 2021. The free text was used to identify included studies including “statin”, “HMG CoA reductase inhibitors”, “non-small cell lung cancer”, “mortality”, “survival”, and “prognosis”, as well as their Medical Subject Headings (MeSH) terms. Two authors independently reviewed the titles and abstracts of these publications according to the inclusion and exclusion criteria, and then the full text was screened for further confirmation. Finally, 16 studies involving 63,273 participants in total were included in our meta-analysis. In addition, an in-house cohort with 101 participants was also included after quality evaluation. The following characteristics of the studies were recorded in Supplemental Table 5: first author’s name, publication year, design, country, case, duration of statin use, and quality score. We performed an additional meta-analysis on the association between the use of statins in patients receiving ICIs and OS. Given that the number of studies in NSCLC was small, we included studies of all kinds of tumors. A total of 6 studies involving 2,135 participants was included, for a total of 2,236 participants following the inclusion of the in-house cohort. The characteristics of the studies were recorded in Supplemental Table 6. HRs and their 95% CIs were recorded to calculate pooled HR using the software Stata 15.0.

### Availability of data and materials.

All data supporting the results in this study are shown in this published article and its supplementary files.

### Statistics.

All statistical analyses were carried out using SPSS 26.0 software or R language 4.0.2. The difference between the 2 groups was analyzed by parametric 2-tailed Student’s *t* test or nonparametric Mann Whitney *U* test, and the difference between multiple groups was analyzed by parametric 1-way ANOVA followed by Tukey’s multiple-comparison test or nonparametric Kruskal-Wallis test followed by Dunn’s multiple-comparison test. All data are presented as mean ± SD of 3 independent experiments, if not noted. Survival analysis was performed by log-rank test. The association between statin use and immunotherapeutic response was performed using Pearson’s χ^2^ test. All statistical tests were 2 sided, and *P* < 0.05 was deemed to be statistically significant.

### Study approval.

Collection of clinical samples in this study was approved by the IRB of The Affiliated Wuxi People’s Hospital of Nanjing Medical University (no. KY21126). All animal experiments were approved by the Laboratory Animal Ethics Committee at Nanjing Medical University.

## Author contributions

MZ, XZ, and JM conceived the study and participated in the study design, performance, coordination, and project supervision. WM, YC, DC, and FW collected the public data and conducted the bioinformatics analysis. DC and YC performed the meta-analysis. JM, WM, GJ, YX, and RC collected the tumor samples and the clinical data. WM, JM, GJ, FW, and XW performed in vitro and in vivo experiments and the IHC staining. WM, YC, and DC wrote the draft. JM,MZ, and XZ revised the manuscript. MZ, XZ, and WM received financial supports. All authors approved the final manuscript.

## Supplementary Material

Supplemental data

Supplemental tables 1-6

## Version 1 08/09/2022

In-Press Preview

## Version 2 09/22/2022

Electronic publication
