# Supplementary material for: Statin shapes inflamed tumor microenvironment and enhances immune checkpoint blockade in non–small cell lung cancer
Source: JCI Insight. 2022 Sep 22;7(18):e161940. doi: 10.1172/jci.insight.161940 (PMC9675559; doi:10.1172/jci.insight.161940)
Supplement: Supplemental data [file jciinsight-7-161940-s077.pdf]

Supplemental Figures

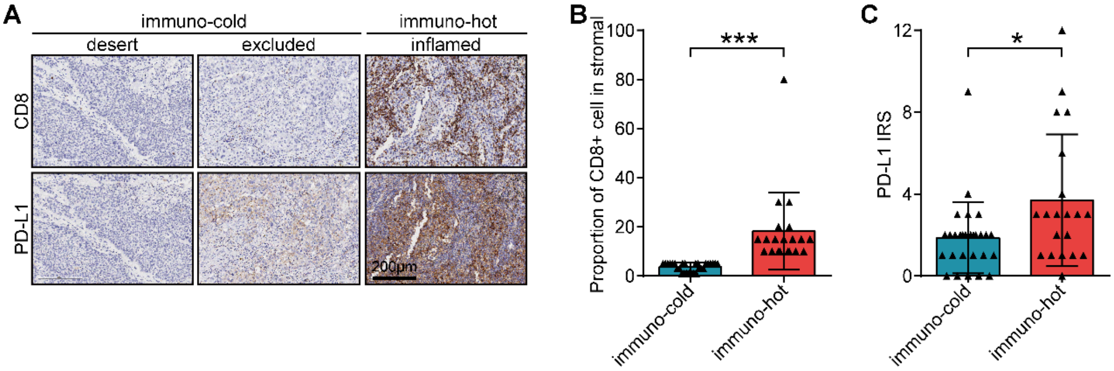

Figure S1. Demarcating the immuno-phenotypes of EGFR-wild NSCLC

(A) Representative images uncovering CD8^{+} T cell infiltration in EGFR-wild NSCLC tissues with different immuno-phenotypes. Magnification: 200×. (B) Infiltrating levels of CD8^{+} T cell in immuno-hot (n=28) and immune-cold (n=20) tumors. Data was presented as mean ± SD. Significance was calculated with Student's t-test. ***P < 0.001. (C) Expression of PD-L1 in immuno-hot (n=28) and immune-cold (n=20) tumors. Data was presented as mean ± SD. Significance was calculated with Student's t-test. *P < 0.05.

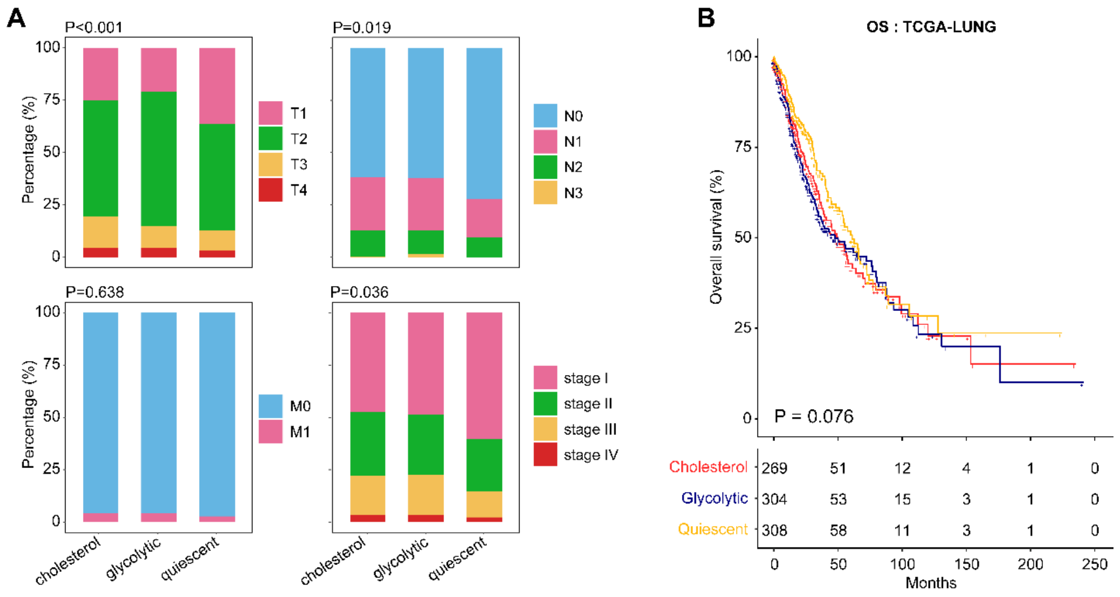

Figure S2. The clinical relevance of metabolic subtypes in EGFR-wild NSCLC

(A) The difference of clinical stages in various metabolic subtypes. Significance was calculated with Pearson's Chi-squared test. (B) The effects of metabolic subtypes on clinical outcomes. Significance was calculated with log-rank test.

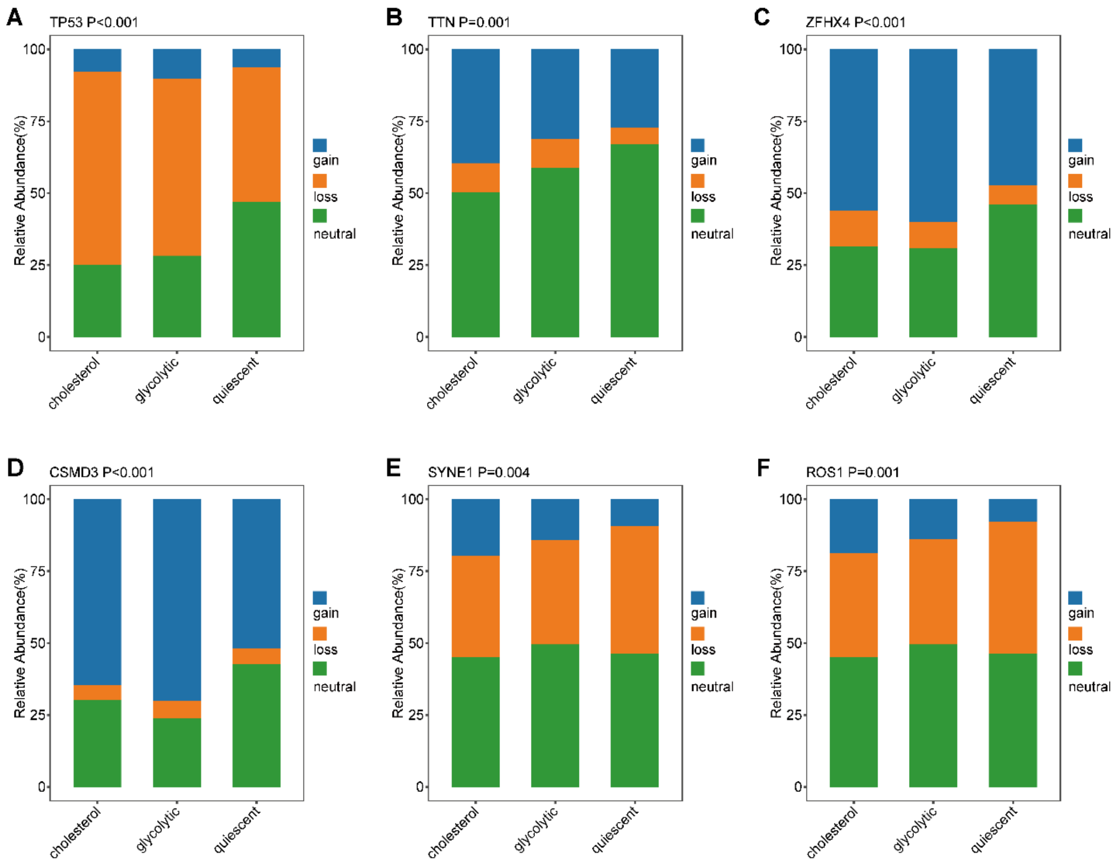

Figure S3. The CNVs of crucial genes in various metabolic subtypes

(A) TP53. (B) TTN. (C) ZFHX4. (D) CSMD3. (E) SYNE1. (F) ROS1. Significance was calculated with Pearson's Chi-squared test.

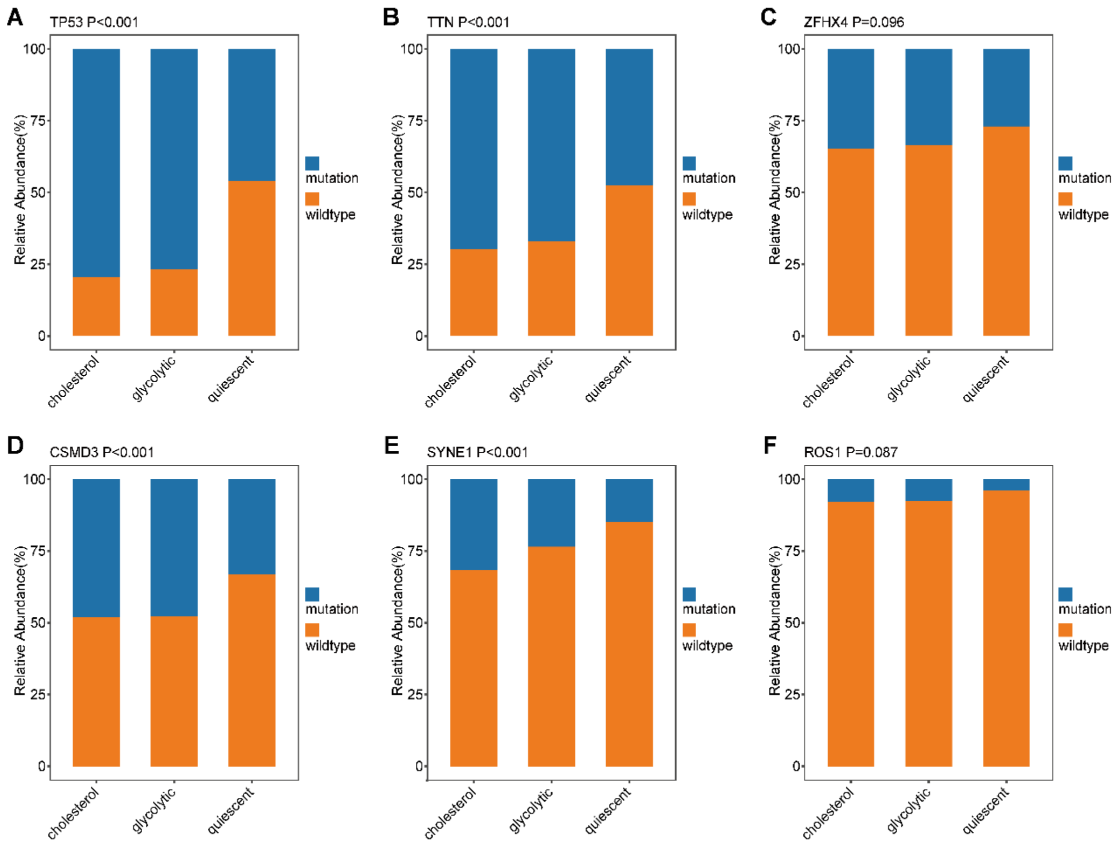

Figure S4. The mutation of crucial genes in various metabolic subtypes

(A) TP53. (B) TTN. (C) ZFHX4. (D) CSMD3. (E) SYNE1. (F) ROS1. Significance was calculated with Pearson's Chi-squared test.

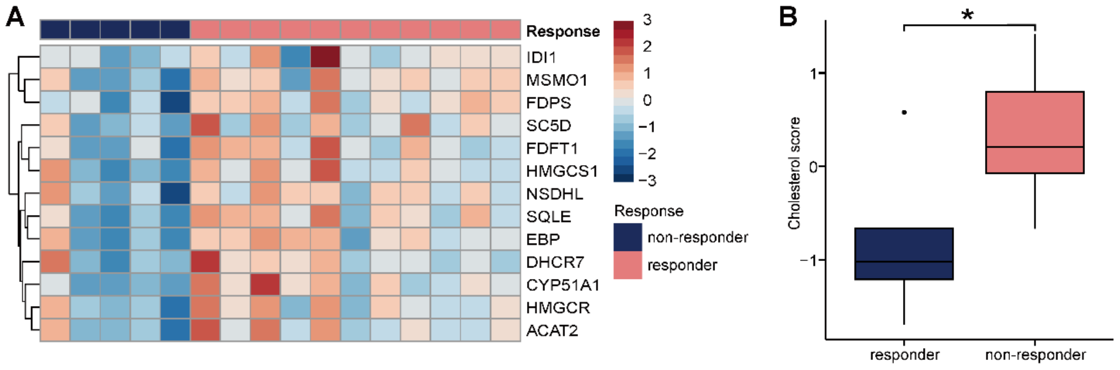

Figure S5. Cholesterol score predicts the response to immunotherapy in NSCLC

(A) Heatmap showing expression levels of coexpressed cholesterol genes in NSCLC samples with different responses to immunotherapy. Data was obtained from the GSE126044 dataset. (B) Box plot showing cholesterol score in responders (n=5) and non-responders (n=11). Data was presented as mean ± SD. Significance was calculated with Student's t-test. *P < 0.05.

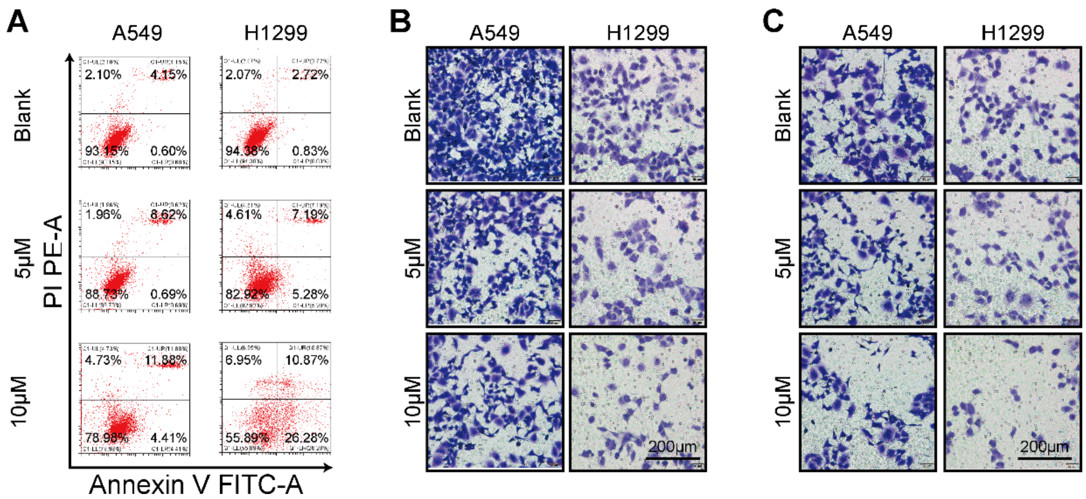

Figure S6. Representative results of cell apoptosis, migration and invasion

(A) The apoptotic levels of NSCLC cells were examined by flow cytometry assay (n = 3). (B, C) The migratory and invasive capacities of NSCLC cells were examined by Boyden chamber assay (n = 3). Magnification: 200×.

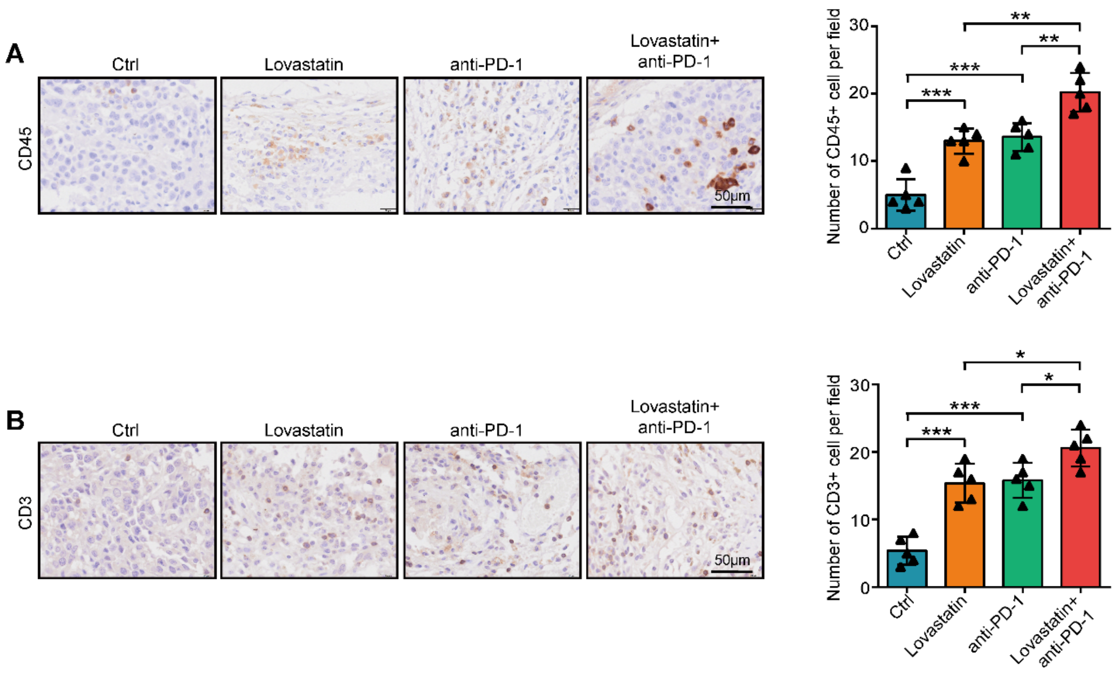

Figure S7. Distribution of CD45+ and CD3+ immune cells in tumors.

Representative images uncovering (A) CD45 and (B) expression in tumor tissues with PBS, lovastatin, anti-PD-1 antibody, and the combination and semi-quantitative analysis (n=5). Magnification: 400×. Data was presented as mean ± SD. Significance was calculated with One-way ANOVA with Tukey’s multiple comparisons test. *P < 0.05, **P < 0.01, ***P < 0.001.

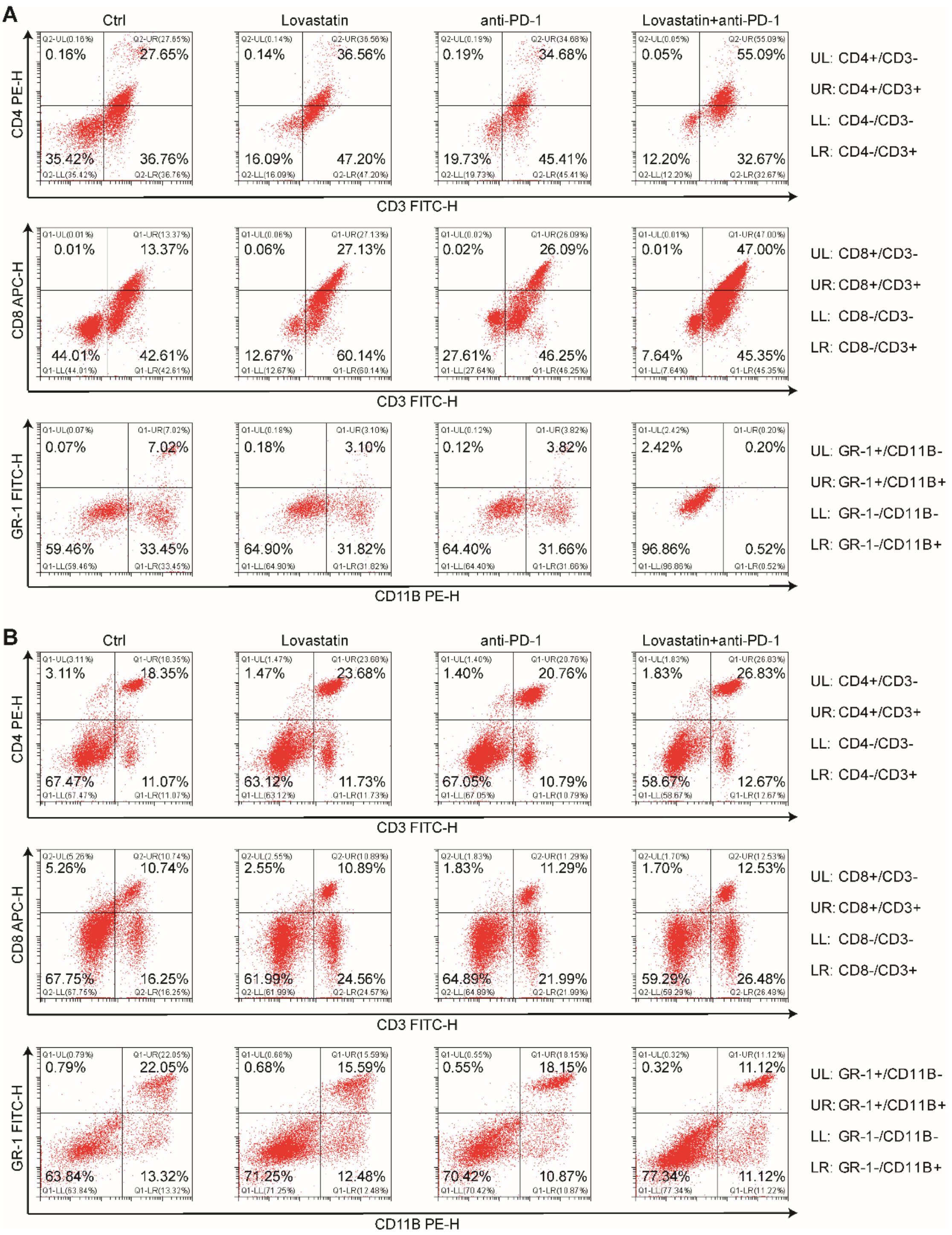

Figure S8. Representative results of flow cytometry analysis (n = 5)

(A) Representative results of flow cytometry analysis of TILs. (B) Representative results of flow cytometry analysis of immune cell subpopulations in the spleen.

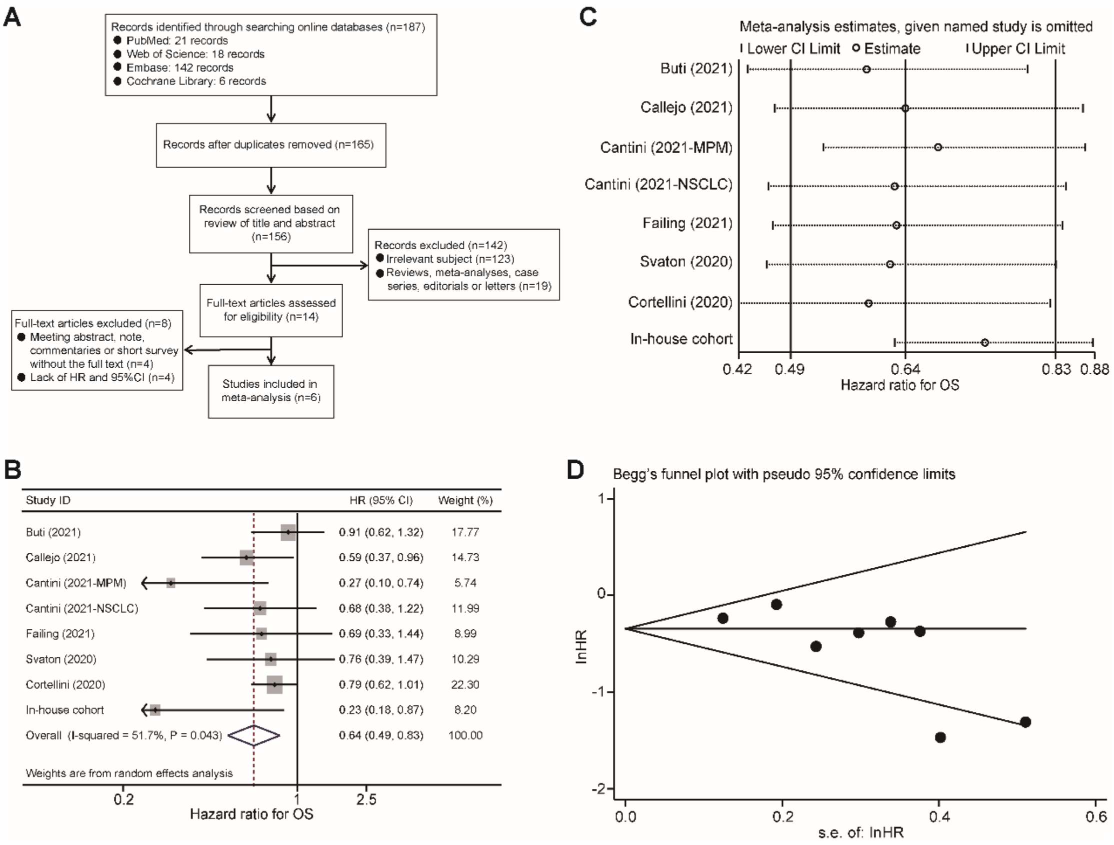

Figure S9. Meta-analysis of statins use and prognosis of patients receiving ICIs

(A) The flow chart of study selection and inclusion. (B) Pooled analysis comparing OS in patients receiving ICIs with statins use compared to those with never statins use. (C) Sensitivity analysis for the association between statins use and OS. (D) Funnel graph regarding the association between statins use and OS.

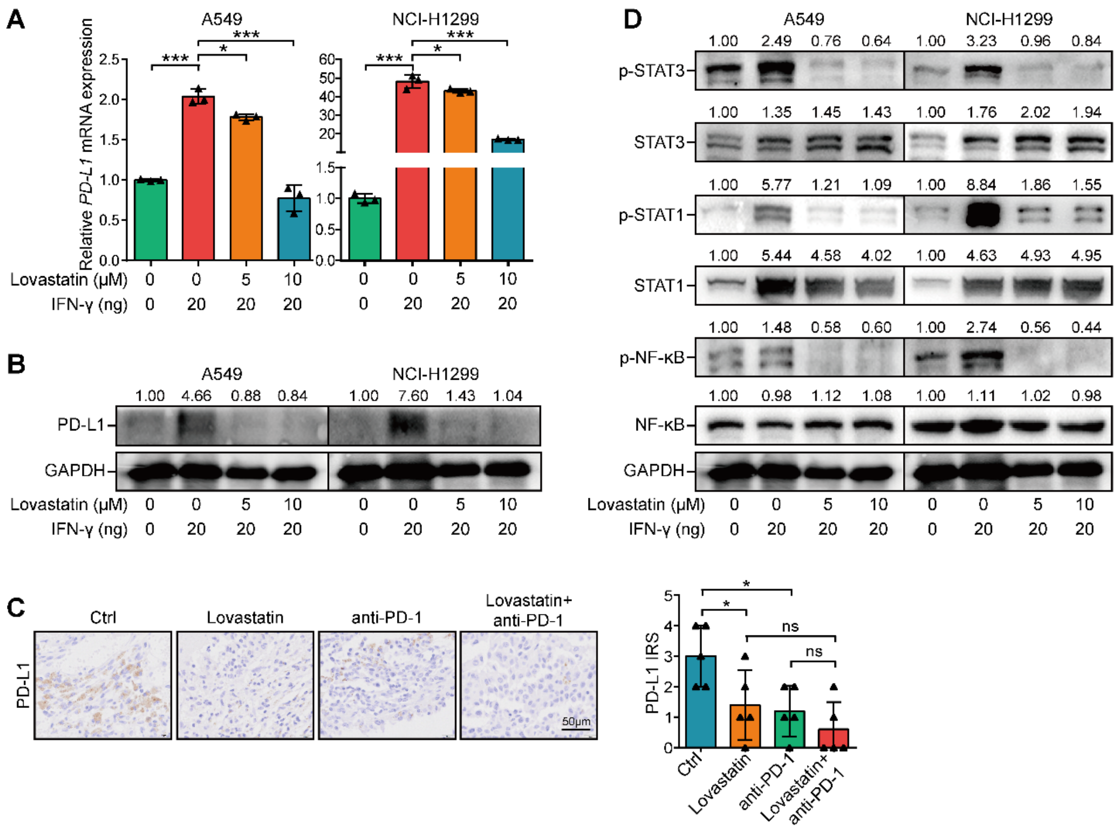

Figure S10. Statin inhibits PD-L1 expression and the phosphorylation of multiple transcriptional factors for PD-L1

(A) The mRNA expression of PD-L1 in control, IFN-γ-induced (20ng/mL, 72 hours), and statin-treated (5/10 μM, 72 hours) serum-starved NSCLC cells were examined by qPCR. The experiment was performed three times. Data was presented as mean ± SD. Significance was calculated with One-way ANOVA with Tukey's multiple comparisons test. *P <0.05, ***P < 0.001. (B) The protein expression of PD-L1 in control, IFN-γ-induced (20ng/mL, 72 hours), and statin-treated (5/10 μM, 72 hours) serum-starved NSCLC cells were examined by western blotting. (C) Representative images uncovering PD-L1 expression in tumor tissues with PBS, lovastatin, anti-PD-1 antibody, and the combination and semi-quantitative analysis (n=5). Magnification: 400×. Data was presented as mean ± SD. Significance was calculated with One-way ANOVA with Tukey's multiple comparisons test. *P < 0.05. (D) The protein expressions of multiple transcriptional factors for PD-L1 in control, IFN-γ-induced (20ng/mL, 72 hours), and statin-treated (5/10 μM, 72 hours) NSCLC cells were examined by western blotting.

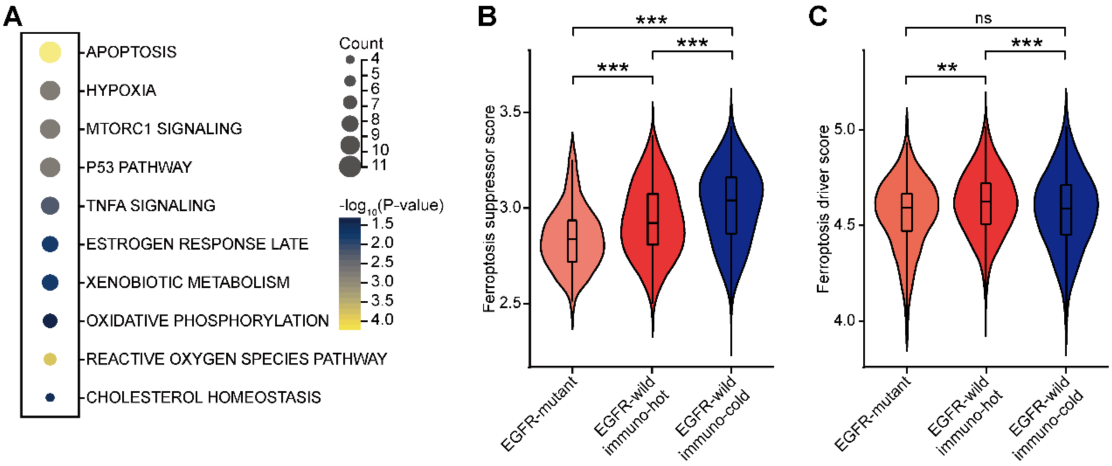

Figure S11. Association between ferroptosis activity and NSCLC subtypes

(A) Dot plot showing Hallmark genesets analysis of ferroptosis suppressors. (B, C) Violin plot showing ferroptosis suppressor and driver scores estimated in EGFR-mutant (n=77), EGFR-wildtype & immuno-hot (n=441), EGFR-wildtype & immuno-cold NSCLC (n=440) groups. Data was presented as mean ± SD. Significance was calculated with One-way ANOVA with Tukey’s multiple comparisons test. **P < 0.01, ***P < 0.001.

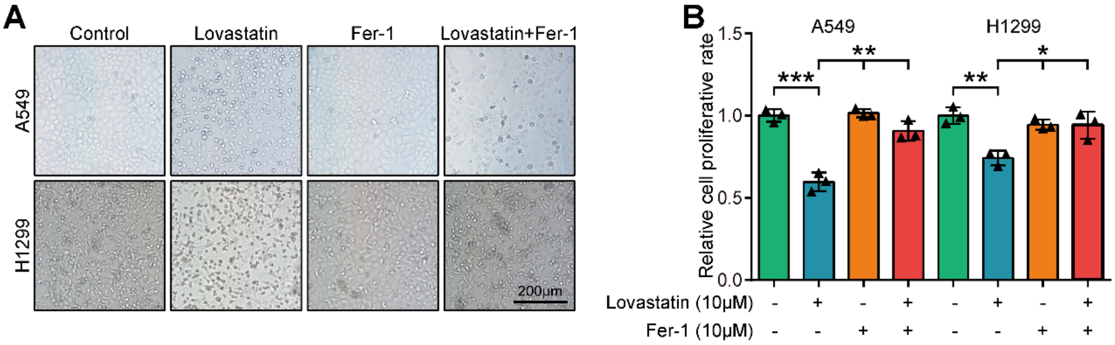

Figure S12. The inhibitor for ferroptosis, fer-1 reverses statin-induced cell death

(A) Representative images showing morphological alterations of control, statin-treated (10 μM, 72 hours), and fer-1-treated (10 μM, 72 hours) NSCLC cells. Statin-treated cells were wrinkled and rounded. Magnification: 200×. (B) The proliferative capacity of control, statin-treated (10 μM, 72 hours), and fer-1-treated (10 μM, 72 hours) NSCLC cells was investigated by CCK-8 assay. The experiment was performed three times. Data was presented as mean ± SD. Significance was calculated with One-way ANOVA with Tukey's multiple comparisons test. *P <0.05, **P < 0.01, ***P < 0.001.
